# Supplementary material for: Optimization of differentiation and transcriptomic profile of THP-1 cells into macrophage by PMA
Source: PLoS One. 2023 Jul 17;18(7):e0286056. doi: 10.1371/journal.pone.0286056 (PMC10351730; doi:10.1371/journal.pone.0286056)
Supplement: S1 File — (ZIP) [file pone.0286056.s001.zip › supporting files/RNA-seq report.zip!/RNA-seq report/5_KEGGEnrichment_result/induce_vs_control/DEG_pathway/hsa04340.html]

hsa04340.png


Close
